# Coparenting Behaviors as Mediators between Postpartum Parental Depressive Symptoms and Toddler’s Symptoms

**DOI:** 10.3389/fpsyg.2016.01912

**Published:** 2016-12-05

**Authors:** Hervé Tissot, Nicolas Favez, France Frascarolo, Jean-Nicolas Despland

**Affiliations:** ^1^Faculty of Psychology and Educational Sciences, University of GenevaGeneva, Switzerland; ^2^Center for Family Studies, Department of Psychiatry, Lausanne University Hospital, University of LausanneLausanne, Switzerland

**Keywords:** maternal depression, paternal depression, coparenting, child symptoms, Lausanne Trilogue Play

## Abstract

Postpartum parental depression, even of mild intensity and short duration, has negative consequences on child development, including increased externalizing and internalizing symptoms. Studies revealed that the links between parental depression and child development are mediated by parenting difficulties. On the other hand, the mediating role of problematic family-level relationships, such as low coparenting support and high conflict between the parents, has rarely been considered, although coparenting difficulties have been linked with both increased depressive symptoms in parents and increased symptoms in toddlers. In the present study, we proposed testing a comprehensive mediation model linking parental depression, coparenting, and child symptoms. At 3 months postpartum, a convenience sample of 69 parental couples completed the Edinburgh Postnatal Depression Scale. In addition, we assessed levels of coparenting support and conflict during a mother–father–infant play situation, the Lausanne Trilogue Play. At 18 months postpartum, both parents assessed child symptoms with the Symptom Checklist Questionnaire. The results showed that coparenting support mediated the links between parental depressive symptoms and child symptoms, but only for mothers: Maternal depressive symptoms were linked with lower coparenting support, which in turn predicted increased psychofunctional symptoms and behavior problems assessed by mothers. Although coparenting conflict behaviors were not predicted by parents’ depressive symptoms, higher conflict was unexpectedly linked with fewer behavior problems assessed by both parents. The present study allowed us to unveil complex pathways between mild parental mood disturbances, family-level relationships, and child development in the first months of the child’s life.

## Introduction

Parental depressive symptoms are common in the postpartum period: Prevalence rates indicate that approximately 15% of mothers and 10% of fathers meet the criteria for clinical depression in the first year postpartum (O’Hara and Swain, 1996; Paulson and Bazemore, 2010). Postpartum depression (PPD) was first considered to be a maternal disorder associated with negative developmental outcomes in children at the social, emotional, and cognitive levels (Lyons-Ruth et al., 2002; Grace et al., 2003; Beebe et al., 2008), including early infant psychofunctional symptoms, such as eating or sleeping difficulties, which can arise as early as 3 months after birth (Righetti-Veltema et al., 2002). Studies showed that the effects of maternal pathology on the child were mainly mediated by impaired maternal parenting capacities and disturbed mother–child relations (for a meta-analysis, see Lovejoy et al., 2000). More recently, the prevalence data for paternal depressive disorders suggested that the postpartum period was also a risk period for fathers, which led researchers to investigate the consequences of paternal PPD. The results globally suggested that paternal PPD could also extensively affect child development (Ramchandani et al., 2005; Goodman et al., 2014) and that fathers’ impaired parenting and relational competences were also likely to mediate these effects (Sethna et al., 2012, 2015; Parfitt et al., 2013). Although researchers have been able to shed light on these processes in both parents, the perspective regarding these phenomena is still fragmented. Indeed, maternal and paternal PPD and its consequences have mostly been studied separately, leading to the construction of dyadic models of the consequences of parental PPD, with linear transmission processes to the child entailed in separate mother–child or father–child relationships. There is now a need to go beyond the parent–child dyads and to adopt a family perspective of the consequences of PPD by investigating the role of family-level relationships in the influence of maternal and paternal PPD on child development.

The role of family-level relationships has rarely been taken into account in the context of parental PPD, although many data suggest that the consequences of parental PPD are not limited to a “depressed parent–child” dyad but can be extended to the whole family system. Indeed, in addition to affecting parent–child dyads, PPD was shown to imply transmission mechanisms within the parent–parent dyad, putting the quality of the parental couple relationship at risk. For example, results showed that PPD is likely to co-occur within families, with higher depression in mothers associated with higher depression in fathers during the whole transition to parenthood (Matthey et al., 2000; Goodman, 2004; Paulson et al., 2006). Moreover, research showed that PPD was likely to be associated with marital distress (Roux et al., 2002; Hanington et al., 2012; Clout and Brown, 2016). These data, indicating that marital dysfunctions are associated with depression—whether as a cause or as a consequence—are crucial, as they suggest the likelihood of another pathway through which PPD might affect the child. Indeed, research on marital distress has extensively shown that marital difficulties, even in the absence of parental depression, may have a major impact on child development (Cummings and Davies, 1994). The “spillover” hypothesis has been formulated to explain the indirect impact of marital conflict on the child (Engfer, 1988). In the case of marital conflict, even when parents try to protect their child from directly witnessing acute emotional outbursts, the negative emotions emerging from the conflict eventually tend to surface during parent–child interactions, with maritally distressed parents being less warm and more rejecting of the child when they interact in a triadic setting (Katz and Gottman, 1996). This spillover effect from the marital to the parent(s)–child subsystems, and therefore the impact of marital distress on the child, has been explained as the intervening effect of family-level variables, such as coparenting (Margolin et al., 2001). For example, marital conflict will likely lead to less support between the parents, which may negatively affect the quality of each parent’s parenting experience. Because PPD was shown to potentially affect the parental couple and because positive coparenting implies coordination between the parents, the role of coparenting needs to be investigated in the context of parental PPD.

Coparenting refers to the coordination between two adults who are rearing a child (or several children) in relation to the child and the child’s education, in relation to the mutual support they give to each other, and in relation to the way in which they work as a team in the rearing tasks (McHale, 1995; Van Egeren and Hawkins, 2004). In intact families with two parents raising one or several children, the marital and the coparenting relationships represent two discrete functions of the couple. Indeed, family systems theory (Minuchin, 1974) has supported the fact that the coparenting relationship should be distinguished from the marital relationship, since both have very different and specific functions for the family members. The marital relationship fulfills the emotional and sexual needs of the parents and exists independently from the children. In contrast, the coparenting relationship “physically” starts from the moment the child arrives in the family—although it may have already emerged during the prepartum period at a psychological level (Altenburger et al., 2014). The coparenting relationship is the executive function through which the parental couple, as a team, provides a secure environment for the child to grow up in. Finally, the coparenting relationship can also exist independently from the marital relationship, such as in divorced couples.

In a different theoretical model, a positive and thus healthy coparenting relationship has been defined as a high level of support and a low level of conflict between the parents. Indeed, high support and low conflict have been repeatedly linked to positive child outcomes, whereas behaviors such as disparagement, criticisms toward the other parent in front of the child, and contradictory parenting behaviors predict lower adjustment in children. Coparenting difficulties were shown to lead to more behavior problems (McHale and Rasmussen, 1998), more depressive and anxious symptoms (Katz and Low, 2004), lower social competencies (Cabrera et al., 2012), or lower performances in Theory of Mind tasks during the school years (Favez et al., 2012). Moreover, a meta-analysis rigorously established an association between coparenting difficulties and externalizing and internalizing symptoms in the child’s first 18 years (Teubert and Pinquart, 2010). Studies in samples of infants are scarcer, but the data available suggested that coparenting difficulties may exert an early impact on the regulation of biological functions, such as sleeping and eating (Cheng et al., 2009; Kim and Teti, 2014).

As PPD was shown to potentially affect the parental couple, it is likely that PPD in one or both parents might affect coparenting, which might in turn affect the child. To date, coparenting has been only rarely taken into consideration in the studies of the consequences of PPD. Recent studies, based on self-reported assessments of coparenting, have reported significant links between both parents’ depressive symptoms, negative coparenting, and negative temperament (Solmeyer and Feinberg, 2011) or poor sleep quality in the child (McDaniel and Teti, 2012). However, self-reported measures of the quality of coparenting behaviors only provide access to each parent’s individual perceptions about these relational phenomena. In comparison, the observation of coparenting behaviors during triadic interactions allow direct access to the interactive behaviors of the coparenting dyad. In a recent study, based on repeated measurement of both parents’ depression and observations of coparenting behaviors during mother–father–child interactions, we found that maternal depression at 3 months was concurrently and longitudinally associated with low coparenting support throughout the first year (Tissot et al., 2016).

The present study specifically aimed to extend these results by testing a mediation model according to which maternal and paternal depressive symptoms would lead to higher coparenting conflict and lower support, which would in turn lead to more negative outcomes in infants, measured in terms of difficult behaviors, and psychofunctional symptoms.

## Materials and Methods

### Participants

We collected data in 69 two-parent families living in the French-speaking part of Switzerland. We recruited families via flyers explaining the context of the research. These flyers were distributed by research assistants at Babyplanet, an annual fair about child care, as well as to every parent visiting the General Register Office and the maternity service of the University Hospital in Lausanne, Switzerland. Socioeconomic status ranged from lower to upper-middle class according to Hollingshead’s two-factor classification, with most fathers and mothers in the upper-middle class (61.2 and 68.2%, respectively), and approximately half of the families with both parents in the upper-middle class (49.2%). At the time of the first meeting for data collection (T1), mothers’ age ranged from 23 to 41 years (*M* = 32.3, *SD* = 4.4) and fathers’ age from 23 to 54 years (*M* = 34.9, *SD* = 5.8). Most of the parental couples were married (75%). Children were 37 boys (54%) and 32 girls (46%). They were mostly first-born babies (68.2%) and were all born healthy and at full-term. At T1, the mean age for children was 98.7 days (*SD* = 9.5). Study inclusion criteria were that (a) both parents and the infant had to live in the same household and that (b) families had to be fluent in French (the language of all testing material).

### Procedure

The longitudinal design of the study included three measurement points at 3 (T1), 9 (T2), and 18 (T3) months postpartum. In the present paper, we focus on data collected at T1 and T3. At T1, we invited the families to our laboratory, where we video recorded the triadic mother–father–infant interactions in the Lausanne Trilogue Play (LTP), a standardized situation of observation (see Measures subsection below). At the end of this session, each parent received a set of self-report questionnaires, including sociodemographic variables, as well as the questionnaire measuring depressive symptoms in parents (see Measures subsection below). These questionnaires had to be filled out separately by each parent within 7 days and returned by mail in postage-paid envelopes. At T3, the parents received another set of questionnaires, including a questionnaire designed to measure the presence and strength of potential symptoms in the child. The instructions for filling out and returning the questionnaires were similar to those at T1.

### Measures

#### Parental Depressive Symptoms

We used the Edinburgh Postnatal Depression Scale (EPDS; Cox et al., 1987) to measure the depressive symptoms in parents. This questionnaire was specifically designed to screen for mothers at risk for PPD, but is now widely used to assess both maternal and paternal PPD (Paulson et al., 2006). The EPDS consists of 10 items that are rated on 4-point scales. The sum of the scores on the 10 items leads to a total score with a maximum of 30 points (α = 0.70 for fathers and α = 0.84 for mothers). The validation study of the French version of the test (Guédeney and Fermanian, 1998) established a score of 11 points as a cutoff for clinical depression; however, we mainly focused on the continuous scores on the scale in the present study.

#### Coparenting

We observed the coparenting behaviors during a mother–father–infant triadic play situation, the LTP (Corboz-Warnery et al., 1993). We ask the parents to play with the child according to a four-part scenario: (a) One parent plays with the child (the active parent role), the other one being “simply present” (the participant-observer role); (b) parents switch roles; (c) the three play together; and (d) parents have a discussion, leaving the child on his or her own for a short while. The infant is placed in a baby chair, which can be oriented in three positions: toward one parent, toward the other, and between the two of them. The chair can be adapted to the postural development of the infant, in a “lay” or “sit” position. The parents sit on regular chairs, with the three chairs positioned and oriented to form an equilateral triangle. We ask the parents to try to play without toys, for example doing what they would do on the changing table. The timing of each part is set at 2 min (with 10 s of transition) and controlled by a research assistant, who gives the parents the signal for the transition from one part to the next by using a light. The timing of the parts has been set according to the naturalistic duration of the classic LTP in less standardized procedures. The order of parts (a) to (c) was counterbalanced to rule out an order effect, and part (d) always occurred last. For coding purposes, the LTPs were videotaped in a multiple-camera technical setting.

We used the Family Alliance Assessment Scales (FAAS; Favez et al., 2011) to assess the quality of the coparenting behaviors observed during the LTP. The FAAS were specifically designed to assess the quality of triadic interactions during the LTP along one categorical scale (global assessment of the “family alliance” typology) and 15 ordinal scales (focused on specific characteristics of the interactions). For the present study, we specifically used two scales: coparenting support and coparenting conflict. The behaviors of the parents are coded as being “Inappropriate,” “Moderate,” or “Appropriate.” Examples of behaviors reflecting appropriate coparenting support are talking positively about the other parent, praising the other parent, encouraging the child to interact with the other parent, thanking the other parent, following the other parent’s lead or previous ideas in the play, showing positive affective expressions when watching the other parent play with the child, and offering material support (e.g., bringing a pacifier if needed). Behaviors indicative of negative support may also occur. Indeed, it has been shown that some maritally distressed parents support each other against the child, thus forming a coalition against the child, who is then put in the position of scapegoat (Minuchin, 1974). These behaviors are considered as negative support. A high score on this scale reflects a high frequency of positive support behaviors and a low frequency of non-appropriate support behaviors. Examples of behaviors indicating high coparenting conflict are competition between the parents, verbal sparring, negative comments to the other parent about his or her behaviors, mockery, negative comments to the child about the other parent, exclusion of a parent by the other, and interference of a parent when the other is playing with the child. A high score on this scale reflects a high frequency of these behaviors. Thus, a high score on the support scale and a low score on the conflict scale indicates positive coparenting.

The following strategy was used to establish interrater reliability. First, an experienced rater coded all the videotapes, while 15 cases were randomly selected and coded by a second rater, who was trained to the coding system by the first rater. We used a weighted kappa statistic to assess interrater reliability, following the recommendations for ordinal data (Norman and Streiner, 2008). The results showed a satisfactory agreement for both scales, according to the recommendations (Landis and Koch, 1977; Hallgren, 2012): Kappas were 0.77 for the coparenting support scale and 0.69 for the coparenting conflict scale. After the computation of interrater reliability, the two coders discussed the discrepancies in the double-coded cases and established a consensus on discrepant codes.

#### Child Symptoms

At T3, both parents completed the Symptom Checklist (SCL; see Robert-Tissot et al., 1989, for the publication of the first version; the unpublished revised version F-95 was used for this study) to assess the presence of psychofunctional and behavioral symptoms in their child. This version of the scale contains 10 items referring to potential symptoms—difficulties falling asleep, waking up during the night, sleeping problems, eating not enough/too much, eating problems, anger crises, opposition/negativity, agitation, attention difficulties, and separation difficulties. Items are rated on 5-point Likert scale ranging from “never” to “often” or from “not characteristic” to “very characteristic”, according to the occurrence of problematic behaviors in the child. One item (item 4: eating not enough/too much) was not used in the analyses, because both extremes of the scale potentially indicated problematic behaviors (1 = “My child does not eat enough,” 5 = “My child eats too much”). This checklist received good predictive validity when non-referred groups of families with children up to 3 years old were compared with families referred for psychofunctional symptoms (Robert-Tissot et al., 1991).

### Statistical Analyses

We performed, as a preliminary analysis, a confirmatory factor analysis on the SCL items by using structural equation modeling (SEM) to reduce the information entailed in the nine items of the scale into a reduced number of factors. This procedure allowed us to increase the statistical power in subsequent analyses. We parceled the items into two dimensions. We specified the model on the basis of theoretical concerns, as SCL contains four items (items 1, 2, 3, and 5) pertaining to the presence of dysregulations of biological functions (sleeping and eating), whereas the remaining five items (items 6–10) refer to problematic externalizing behaviors. Thus, we tested a two-factor model, with items 1, 2, 3, and 5 loading on one factor, namely, the psychofunctional symptoms factor, and items 6–10 loading on the second factor, namely, the externalizing symptoms factor. Moreover, both factors were allowed to covary. Measurement invariance (MI) between mothers and fathers was also tested. MI requires the same model to be tested across different groups in order to examine whether the links between latent and observed variables differ from one group to another. Three nested models with increased degrees of constraint were compared in multigroup analyses (fathers versus mothers): We specified a first model of configural invariance, in which the parameters (factor loadings, item intercepts, residual variances, factor variances, and covariance) were freely estimated in each group, whereas the factor means were constrained to zero in both groups. We then tested metric invariance, in which we added equivalence constraints on the factor loadings across the two groups. In a third model, we tested scalar invariance, in which equivalence constraints were imposed on factor loadings and on the item intercepts, while the factor means were constrained to zero in one group and freely estimated in the other group.

After these preliminary analyses, we conducted a full set of descriptive analyses, including means, and standard deviations for all the variables under study. Finally, in the main set of analyses, we tested the adjustment of a mediation model, with maternal and paternal depression predicting coparenting support and conflict, which in turn predicted child symptoms assessed by both parents. The adjustment of this mediation model was also estimated with SEM techniques. Estimation of mediation effects was performed by using the Monte Carlo method for assessing mediation (MacKinnon et al., 2004; Selig and Preacher, 2008; Preacher and Selig, 2012), which consisted of examining Monte Carlo confidence intervals (CIs) of indirect effects. The CI level was set to 95% and the simulation was repeated 20,000 times.

All the models were estimated by using a full information maximum likelihood estimator. Besides the chi-square significance, the adjustment of the models was estimated according to the standard criteria defined by Hu and Bentler (1999). For the comparative fit index (CFI), values below 0.90 indicate a poor fit of the model, values above 0.90 a fair fit, and values above 0.95 an excellent fit. For the root mean square error of approximation (RMSEA), values above 0.08 indicate a poor fit of the model, values between 0.08 and 0.06 a fair fit, and values below 0.06 an excellent fit.

We performed the descriptive analyses with IBM SPSS Statistics 23 software and SEM analyses with IBM SPSS Amos 23 software, and we computed the Monte Carlo CIs in R.

## Results

### Preliminary Analyses: SCL Factor Structure

The preliminary analyses consisted of testing a two-factor factor structure for the SCL and testing for MI across fathers and mothers. The results for the first model, which assumed configural invariance between mothers and fathers, globally showed good adjustment of the model (**Table 1**). The metric and the scalar model showed even better fit. Likelihood ratio tests showed that the scalar model should be preferred, as its adjustment was not statistically poorer than either the configural (χ^2^ = 5.979, *df* = 16, *p* = 0.988) or the metric model (χ^2^ = 4.858, *df* = 9, *p* = 0.847). This result showed that, for both mothers and fathers, all responses to the items in the test were explained by a similar two-factor structure, that the strength of the links between latent and observed variables was similar for both parents, and that the intercepts of the observed variables could be considered equivalent. In other words, the two factors of psychofunctional symptoms and externalizing symptoms were shown to be relevant for both mothers and fathers and could be derived in a similar way for both parents from their responses to the items. Thus, for each parent, we computed one index of severity of psychofunctional symptoms by averaging the scores on items 1, 2, 3, and 5 and one index of externalizing symptoms by averaging the scores on items 6–10. These indices were used as observed variables in the subsequent analyses.

**Table 1:** Adjustment of the two-factor model of the Symptom Checklist (SCL) across different levels of measurement invariance between mothers and fathers.

						90% CI	
	χ^2^	*df*	*p*	CFI	RMSEA	LL	UL
**Invariance**							
Configural	67.879	52	0.069	0.912	0.047	0.000	0.076
Metric	69.002	59	0.175	0.945	0.035	0.000	0.066
Scalar	73.858	68	0.293	0.968	0.025	0.000	0.058

*CFI, comparative fit index; RMSEA, root mean square error of approximation; CI, confidence interval; LL, lower limit; UL, upper limit.*

### Descriptive Statistics

Descriptive statistics for all study variables can be found in **Table 2**. Concerning parental depressive symptoms, results showed that mean scores for both mothers and fathers were lower than the cutoff for clinical depression, although 14 women (21.5%) and 6 men (9.2%) obtained scores higher than the cutoff for clinical depression. A paired-sample *t-*test showed that mothers, on average, were more depressed than fathers, *t*(61) = 2.78, *p* < 0.01. Concerning coparenting, as expected in a convenience sample of intact families, the average level of support was in the higher range, with 42 families (61.8%) obtaining the highest support score, and only four families (5.9%) obtaining the lowest score. As expected, the average score for conflict was in the lower range of the scale, and most families (*n* = 36, 52.9%) obtained the lowest score on the conflict scale, whereas only three families (4.4%) showed the highest level of coparenting conflict during the triadic play. Concerning infants’ symptoms, paired-sampled *t-*tests did not reveal any differences between mothers’ and fathers’ reports, concerning either psychofunctional symptoms, *t*(59) = 0.38, *p* = 0.97, or externalizing symptoms, *t*(59) = 1.07, *p* = 0.29.

**Table 2:** Descriptive statistics for self-reported parental depressive symptoms, observed coparenting, and child’s symptoms reported by parents.

		Range			
	*N*	Min.	Max.	*M*	*SD*
**Self-reported depressive symptoms**					
Mothers	65	0.00	24.00	6.72	4.61
Fathers	65	0.00	15.00	5.09	3.19
**Observed Coparenting**					
Support	68	0.00	2.00	1.56	0.61
Conflict	68	0.00	2.00	0.51	0.59
**Child’s psychofunctional symptoms**					
Mothers’ report	60	1.00	3.50	1.87	0.66
Fathers’ report	60	0.00	3.50	1.87	0.75
**Child’s externalizing symptoms**					
Mothers’ report	60	1.20	4.80	2.63	0.72
Fathers’ report	60	0.00	4.20	2.51	0.82

### Mediational Model

**FIGURE 1:**
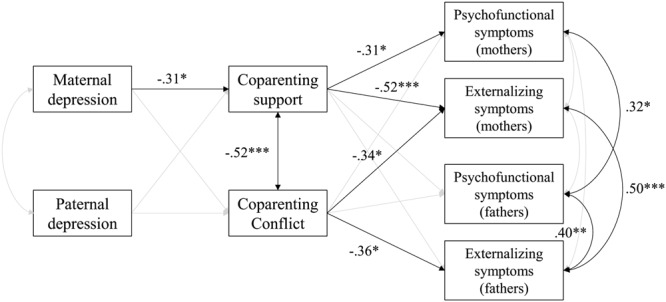
**Coparenting support and conflict as mediators between maternal and paternal depressive symptoms and child symptoms.** Model fit: χ^2^ = 9.361, *df* = 8, *p* = 0.313, CFI = 0.983, RMSEA = 0.050, 90% CI [000, 0.156]. Non-significant paths appear in lighter gray. Displayed parameters are standardized coefficients. ^∗^*p* < 0.05, ^∗∗^*p* < 0.01, ^∗∗∗^*p* < 0.001.

The main analyses consisted of testing a model in which coparenting support and conflict mediated the links between maternal and paternal depression and child psychofunctional and externalizing symptoms (**Figure 1**). Results of the estimation of this model showed that it had an excellent fit, χ^2^ = 9.361, *df* = 8, *p* = 0.313, CFI = 0.983, RMSEA = 0.050, 90% CI [0.000, 0.156].

The parameter estimation showed that higher depressive symptoms in mothers, but not in fathers, predicted lower coparenting support. On the other hand, neither maternal nor paternal depressive symptoms significantly predicted coparenting conflict. Concerning the links between coparenting and infants’ symptoms, results showed that lower coparenting support predicted higher psychofunctional and externalizing symptoms, but only when assessed by mothers. Higher coparenting conflict, surprisingly, predicted lower externalizing symptoms assessed by both parents. Concerning the covariance between the variables, coparenting support was significantly and negatively related to coparenting conflict. Maternal and paternal reports of psychofunctional symptoms in the child were significantly correlated. A similar link appeared for externalizing symptoms. Moreover, fathers who reported higher psychofunctional symptoms in their child also reported more externalizing symptoms. Finally, higher maternal depressive symptoms were, surprisingly, not significantly related to paternal depressive symptoms.

According to these results, two mediation paths were of particular interest, as higher maternal depressive symptoms predicted lower coparenting support, which predicted higher psychofunctional and externalizing symptoms. Concerning the first path linking higher maternal depressive symptoms to higher psychofunctional symptoms assessed by mothers via a decrease in coparenting support, the unstandardized indirect effect was (-0.040)(-0.344) = 0.014 and the standardized indirect effect was (-0.309)(-0.314) = 0.097. The estimation of Monte Carlo CIs showed that this effect could be considered significant, as the unstandardized CIs did not comprise the value of 0, 95% CI [0.001, 0.034]. Concerning the second path linking higher maternal depressive symptoms to higher externalizing symptoms assessed by mothers mediated by lower coparenting support, the unstandardized indirect effect was (-0.040)(-0.625) = 0.025 and the standardized indirect effect was (-0.309)(-0.517) = 0.160. The estimation of Monte Carlo CIs showed that this effect was also significant, as the unstandardized CIs did not comprise the value of 0, 95% CI [0.005, 0.053].

## Discussion

The aim of this study was to test the hypothesis that the impact of maternal and paternal depressive symptoms on the child might be mediated by coparenting support and conflict. We hypothesized that, at 3 months postpartum, higher maternal and paternal depressive symptoms would lead to lower support and higher conflict between the parents during triadic interactions, which would increase the probability of the presence of psychofunctional and externalizing symptoms 15 months later. The results globally indicated that only coparenting support was a significant mediator. Moreover, only the links between depressive symptoms in mothers at 3 months postpartum and their own report of the presence of symptoms in the child 18 months later was mediated by coparenting support. In contrast, we did not find any association between maternal symptoms and coparenting conflict, nor did we find any effects for fathers, whose level of depression was not related to either coparenting support or conflict. These results thus allowed us to only partially confirm our hypothesis.

The most relevant finding of the present study, according to which higher maternal depressive symptoms would lead to lower coparenting support, which would lead to higher symptoms in the child, is in line with the results of previous studies in the field. It bridges the gap between two separate lines of study, the first one suggesting the links between parental depression and family-level relationships (Dickstein et al., 1998; Solmeyer and Feinberg, 2011; Tissot et al., 2016) and the second one repeatedly demonstrating the unique impact of coparenting on child development (e.g., Teubert and Pinquart, 2010). The present finding suggests that, first, the more depressed a mother is, the less the parents will show support during a triadic task. It is likely that a mother presenting depressive symptoms will have a hard time supporting her partner when they are together with their child. She may use her partner as a support if she experiences a few depressive symptoms and rests on her partner’s shoulders. In the long run, this may quickly relieve her and eventually help her to recover from her depressive symptoms, but it might also put much pressure on the father, increasing the risk, in turn, of him also being overwhelmed by too many responsibilities in the family and later developing depressive symptoms. Following the family systems theory, we assume that coparenting support is necessarily a mutual process and that a parent in need of support may struggle to support his or her partner in return. Nevertheless, the present study did not reveal similar processes for fathers, suggesting that fathers’ depressive symptoms might not impact family-level relationships, or the child, as strongly as maternal symptoms do. It is likely that the greater impact of maternal depressive symptoms might be because mothers are still the primary caregivers in most families in Western countries. In Switzerland, where we conducted the study, most fathers in middle to high socioeconomic status families work full time—or close to full time—whereas the mothers at 3 months postpartum are mostly still on maternity leave, which may explain why a father’s depressive state is less closely related to family functioning than is that of the mother in the first months, especially in cases of a mild or moderate depressive state, as we were able to measure in the present low-risk sample. It is likely that studies of higher risk samples might lead to different conclusions. Still, the present study suggested that, at 3 months postpartum, mild depressive symptoms in mothers, but not in fathers, might jeopardize the development of a healthy and positive coparenting relationship between the parents.

Besides the links between maternal depressive symptoms and coparenting support, the existence of mediation effects suggested that lower coparenting support was linked to higher psychofunctional and externalizing symptoms reported by mothers. This result was not surprising, as a non-supportive coparental dyad may represent an insecure environment for a child to grow up in (Cummings et al., 2006), which may be the cause of difficult behaviors and disturbances in biological functions in the child (McDaniel and Teti, 2012). To date, most studies in this field have been conducted in samples of families with school-aged children or adolescents. The present study is one of the few to document links between coparenting difficulties observed during triadic interactions and early child symptoms in the first 2 years of life. The fact that the increase in child symptoms following difficulties in coparenting support was not corroborated by fathers’ reports of child symptoms might be because mothers, as primary caregivers, are more able than fathers to detect potential symptoms in their child. However, we still need to be cautious in the interpretation of these results, as they may partly be explained by mothers’ biased perceptions of their child. Indeed, depressed mothers have been shown to assess their child’s behaviors and outcomes more severely than an external observer does (Field et al., 1993). At this point, we cannot exclude the possibility that the increased symptoms reported in their child by mothers in the higher range of depressive symptoms in this sample was, in fact, because of the mothers’ biased perceptions of their child. Thus, in a further study, it will be necessary to have an external observer collect data about child outcomes in order to control for this potential bias.

Contrary to the results about the mediation effect of coparenting support, the presence or absence of parental conflict did not mediate the link between parental depression and child symptoms. However, higher coparenting conflict was, surprisingly, related to lower externalizing symptoms in the child assessed by both parents. Although this result was unexpected, different explanations can be considered: First, we need to highlight that, in this low-risk sample, the higher rates of coparenting conflict behaviors, such as competitive behaviors, were rarely associated with increased levels of aggressiveness or negative emotions between the parents. We could presume that a moderate level of interparental conflict, in the absence of acute negative emotions, is a sign of increased involvement by both parents. In contrast, a low level of conflict might indicate that one parent is withdrawn from the triad, which has been associated with more negative child outcomes (Favez et al., 2006). Second, in line with the results of other studies that unexpectedly found a negative association between triadic coordination and marital satisfaction in parents (Favez et al., 2011), it is likely that parents in a more positive family context—e.g., with a lower level of coparenting conflict—might be more able to develop reflexive thinking and thus be more critical in their assessment of child symptoms, whereas parents facing difficulties within the family might trigger defense mechanisms, such as denial, leading to a bias in the assessment of their child in a falsely positive way. Finally, as the assessment of child behaviors was done by the parents themselves and did not imply any clinical assessment of the severity of child symptoms, we should consider that an increase in externalizing symptoms was not necessarily negative, especially since both mothers and fathers rarely rated the symptoms in the upper range of the scale. Indeed, it seems reasonable that a child might sometimes show anger, opposition, negativity, agitation, attention difficulties, or separation difficulties, without considering that this would necessarily indicate maladjustments. We insist that these results have to be cautiously interpreted until they are confirmed in a replication study, in which an external observer collects the data about the child.

The present study had some limitations. First, the sample size was modest, which might have been due to the methodological complexity of this study, as the study design required a heavy time commitment by the families (observational data, longitudinal design, and measurement of variables in both parents together). The sample size might have limited the generalizability of our findings and have increased Type II errors, which might partly explain the lack of significant findings concerning fathers. An effort should be made to replicate these findings in larger samples. Further studies might also be conducted in samples of parents presenting more severe depression or globally presenting more risk factors associated with PPD. Although such studies will be difficult to conduct, especially in terms of recruitment and selection criteria, they would certainly help to extend our knowledge about the links between parental psychopathology, family-level processes, and child adjustment.

Despite these limitations, the present study allowed us to unveil complex pathways between mild maternal mood disturbances, family-level relationships, and child development in the first months of the child’s life. It stressed, once again, the importance of family researchers observing the family functioning *in vivo* and going beyond the mother–child dyad to widen our understanding of the relational processes within the family. To this end, it is necessary to use the observation of the mother–father–child triadic interaction as an index for measuring the quality of family-level relationships, whose relevance for understanding individual functioning, and in particular, child development, is now irrefutable and thus can no longer be called into question.

## Ethics Statement

This research protocol had been accepted by the review board of the ethical committee of the Lausanne University Hospital. The participants and the researchers signed a consent form, specifying that the participants can withdraw from the study and their data be deleted upon request at any time.

## Author Contributions

NF, FF, and J-ND designed the study described in the manuscript and supervised the data collection. HT participated in the data collection, performed the analyses, and wrote the first draft of the paper. The first draft of the paper was then carefully revised by NF, FF, and J-ND.

## Conflict of Interest Statement

The authors declare that the research was conducted in the absence of any commercial or financial relationships that could be construed as a potential conflict of interest.
